# Alveolar type II cell therapy ameliorates pulmonary fibrosis by reprogramming macrophage activation

**DOI:** 10.1186/s41232-026-00434-4

**Published:** 2026-07-10

**Authors:** Raquel Guillamat-Prats, Gemma Gay-Jordi, Paula Duch, Sabrina Gea-Sorlí, Luis Ignacio Sanchez-Lopez, Victor Ivo Peinado, Anna Serrano-Mollar

**Affiliations:** 1 Department of Experimental Pathology, Institut d’Investigacions Biomèdiques de Barcelona, CSIC-IDIBAPS, C/Rosselló, 161, 7ª, 08036 Barcelona, IIBB Spain; 2 Biomedical Research Networking Centre in Respiratory Diseases (CIBERES), Madrid, Spain; 3 Respiratory and Immune Repair Group (REPAIR), Germans Trias I Pujol Research Institute (IGTP), Badalona, Spain; 4 Biogipuzkoa (Previosly Known As Biodonostia) Health Research Institute, San Sebastian, Spain; 5 Department of Pulmonary Medicine, Hospital Clinic-IDIBAPS, Barcelona, Spain

**Keywords:** Alveolar Type II cells, Pulmonary fibrosis, Macrophage reprogramming, Cell therapy, Fibroblast activation, CXCL12/CXCR4 axis, Paracrine signalling

## Abstract

**Background:**

Alveolar type II (ATII) cell transplantation is a promising therapeutic strategy for idiopathic pulmonary fibrosis, although its underlying cellular mechanisms remain incompletely understood. Given that macrophage profibrotic activation is a key driver of fibrosis, we hypothesized that ATII cell therapy exerts antifibrotic effects by reprogramming macrophage activation states during the established fibrotic phase.

**Methods:**

In a rat model of bleomycin-induced pulmonary fibrosis, we characterized alveolar and interstitial macrophage profiles in control animals, in fibrotic animals, and after intratracheal transplantation of ATII cells performed on day 15 after bleomycin once fibrosis was established. Gene expression analyses were used to define macrophage activation profiles, while in vitro co-cultures of macrophages with ATII cells or fibroblasts assessed paracrine interactions and macrophage activation-state modulation. The CXCL12/CXCR4 signalling axis was further examined to explore potential mechanisms linking macrophage reprogramming to CXCR4⁺ cell dynamics, including recruitment of circulating CXCR4⁺ cells.

**Results:**

Disease progression promoted a prominent profibrotic activation profile enriched in M2-associated markers in both alveolar and interstitial macrophages. ATII cell transplantation markedly reduced macrophage infiltration and shifted their activation toward a less profibrotic, more homeostatic profile. In vitro, soluble mediators released by healthy ATII cells contributed to modulation of macrophage activation states, restoring a less profibrotic activation profile. Reciprocal crosstalk between macrophages and fibroblasts was observed: fibrotic macrophages enhanced fibroblast activation, whereas macrophages from control lungs exerted antifibrotic effects. Notably, ATII cell transplantation normalised CXCL12/CXCR4 axis expression and reduced the number of circulating CXCR4⁺ cells, suggesting that modulation of this axis may limit the recruitment of circulating CXCR4⁺ cells and attenuate profibrotic signalling.

**Conclusions:**

ATII cell transplantation ameliorates pulmonary fibrosis by restoring a less profibrotic immune microenvironment and modulating macrophage-fibroblast crosstalk. These effects are associated with reduced fibroblast activation and decreased accumulation of circulating CXCR4⁺ cells, consistent with attenuation of fibrotic responses.

**Supplementary Information:**

The online version contains supplementary material available at https://doi.org/10.1186/s41232-026-00434-4.

## Introduction

Idiopathic pulmonary fibrosis (IPF) is a progressive and fatal interstitial lung disease characterized by excessive extracellular matrix accumulation, alveolar epithelial cell injury, and aberrant tissue remodelling [1, 2]. Despite advances in antifibrotic therapies, median survival remains limited to approximately 3–5 years from diagnosis, reflecting the urgent need for novel therapeutic approaches. Current approved antifibrotic drugs, nintedanib and pirfenidone, have demonstrated efficacy in slowing functional decline but do not halt disease progression or reverse established fibrosis [3–5].

Pathophysiologically, IPF involves persistent alveolar epithelial damage and impaired regeneration, with depletion of both type I and type II alveolar epithelial cells (ATI and ATII) contributing to progressive epithelial dysfunction [5–7]. ATII cells, which serve as progenitors of ATI cells, are essential for alveolar renewal and repair. Damaged ATII cells not only limit epithelial repair but also create a proinflammatory and profibrotic pulmonary niche by inducing macrophage activation [8–10].

Pulmonary macrophages are the most abundant resident immune cells in both healthy and fibrotic lungs, characterized by remarkable plasticity and the ability to adopt diverse activation states in response to microenvironmental signals [11–14]. Although the M1/M2 framework provides a useful operational model to describe macrophage activation states, pulmonary macrophages in fibrosis display substantial phenotypic heterogeneity and dynamic transitional states that extend beyond this simplified classification. Within this framework, classically activated M1-associated macrophages produce pro-inflammatory mediators, including interleukin-1β (IL-1β) and tumor necrosis factor α (TNF-α), which dominate early injury responses. Alternatively activated and reparative M2-associated macrophages secrete profibrotic factors such as transforming growth factor β (TGF-β), interleukin-10 (IL-10), arginase-1 (Arg-1), and mannose receptor (MR), promoting extracellular matrix deposition and tissue remodelling [9, 15–17].

During IPF progression, macrophages display dynamic heterogeneity but frequently acquire profibrotic activation states enriched in M2-associated markers, driving fibroblast activation, myofibroblast differentiation, and excessive matrix deposition. Advances in single-cell sequencing have revealed distinct macrophage subpopulations enriched in oxidative phosphorylation pathways that sustain profibrotic activity [14, 18, 19]. Both alveolar macrophages (AM) and interstitial macrophages (IM), which include resident and monocyte-derived populations, contribute to fibrogenesis through crosstalk with fibroblasts and fibrocytes. This interaction drives tissue stiffening and loss of lung function [14].

Recruitment of CXCR4⁺ cells, including fibrocytes and other hematopoietic populations, is largely mediated through the CXCL12/CXCR4 axis, which is upregulated in IPF lungs and has been implicated in fibroblast proliferation, migration, and extracellular matrix deposition [20, 21]. Pharmacological inhibition of CXCR4 experimentally attenuates fibroblast activation and fibrogenesis, underscoring this pathway as a potential therapeutic target [14, 21].

Translational studies in bleomycin (BLM)-induced pulmonary fibrosis models demonstrate that intratracheal transplantation of healthy ATII cells restores epithelial integrity, reverses fibrogenesis, and recovers surfactant protein expression, further supporting the pivotal role of these cells in tissue repair [22, 23]. These effects extend to enhanced alveolar biomechanics and improved lung function [24]. Transplantation of iPSC-ATII-like cells mimics these benefits, decreasing fibrosis and collagen deposition through regulation of TGF-β and α-SMA [25]. Notably, a clinical pilot study confirmed that ATII cell therapy is safe, well tolerated, and may stabilize disease progression while improving patient quality of life [26].

Emerging data indicate that the antifibrotic actions of ATII cells involve paracrine signalling to modulate macrophage activation states and function [27–29]. Accordingly, shifting macrophage activation away from a profibrotic state may attenuate fibrotic progression. The exact molecular mechanisms underlying this crosstalk remain an important area for investigation.

Given the central role of macrophages in fibrogenesis and the modulatory potential of ATII cells, we hypothesize that ATII cell transplantation ameliorates fibrosis by restoring epithelial homeostasis as well as reprogramming macrophage activation in the lung. Here, we investigate the effect of ATII cell transplantation on the phenotype of alveolar and interstitial macrophages and evaluate how these macrophage changes influence downstream fibroblast activation. In addition, we assess the CXCL12/CXCR4 axis as a complementary mechanism potentially linking macrophage reprogramming with the recruitment of circulating CXCR4^+^ cells and fibrogenesis.

## Methods

### Animals

Male Sprague–Dawley rats (Charles River, France), weighing 200–225 g and aged approximately 8–10 weeks, were used in compliance with the European Community Directive 86/609/EEC and Spanish guidelines for the care and use of experimental animals. The study was conducted and reported in accordance with the ARRIVE 2.0 guidelines for reporting animal research. Animals were housed under standard laboratory conditions, with controlled temperature (22 ± 2 °C), humidity (55 ± 10%), and a 12 h light/dark cycle, with food and water available ad libitum. Environmental enrichment (red methacrylate tubes, wooden chew sticks, and food pellets placed inside the cage) was provided as part of standard housing.

### Cell isolation

#### Macrophages

Alveolar macrophages (AM) and interstitial macrophages (IM) were isolated from lungs of control and bleomycin (BLM)-treated rats. Throughout the manuscript, BAL-derived macrophage-enriched populations are operationally referred to as alveolar macrophages (AM), whereas post-BAL lung tissue-derived macrophage-enriched populations are referred to as interstitial macrophages (IM). These terms are used for simplicity throughout the manuscript; however, particularly under fibrotic conditions, these populations are unlikely to represent pure resident macrophage subsets and may include infiltrating monocyte-derived macrophages.

AM were obtained by bronchoalveolar lavage (BAL): lungs were lavaged five times with 10 mL aliquots of sterile saline (0.9% NaCl) via tracheal cannula. BAL fluid was centrifuged at 500 × g for 10 min at 4 °C. The cell pellet was resuspended in RPMI-1640 medium supplemented with 10% fetal calf serum, 2 mM glutamine, 100 U/mL penicillin, and 100 μg/mL streptomycin, and cultured for 2 h to allow adherence. Non-adherent cells were removed, and adherent cells were trypsinized for subsequent co-culture and gene expression analyses.

IM were isolated from lung tissue after BAL by enzymatic digestion: lungs were incubated in 0.25% trypsin (Sigma-Aldrich, Spain) diluted in 60 mL sterile saline at 37 °C for 30 min, chopped into 1–2 mm^2^ fragments, and treated with DNase (Roche, Spain) (7500 U/100 mL saline) to degrade extracellular DNA released during tissue digestion, reducing cell clumping. The cell suspension was filtered through nylon meshes (150–30 µm) and centrifuged at 500 × g for 20 min at 20 °C over a sterile Percoll gradient (GE Healthcare, USA) to enrich for monocyte-containing fractions. Following a second DNase treatment (2000 U/100 mL) to further minimize extracellular DNA, cells were plated in supplemented RPMI-1640 medium, and adherent IM were collected after 2 h by trypsinization.

**Fig. 1:**
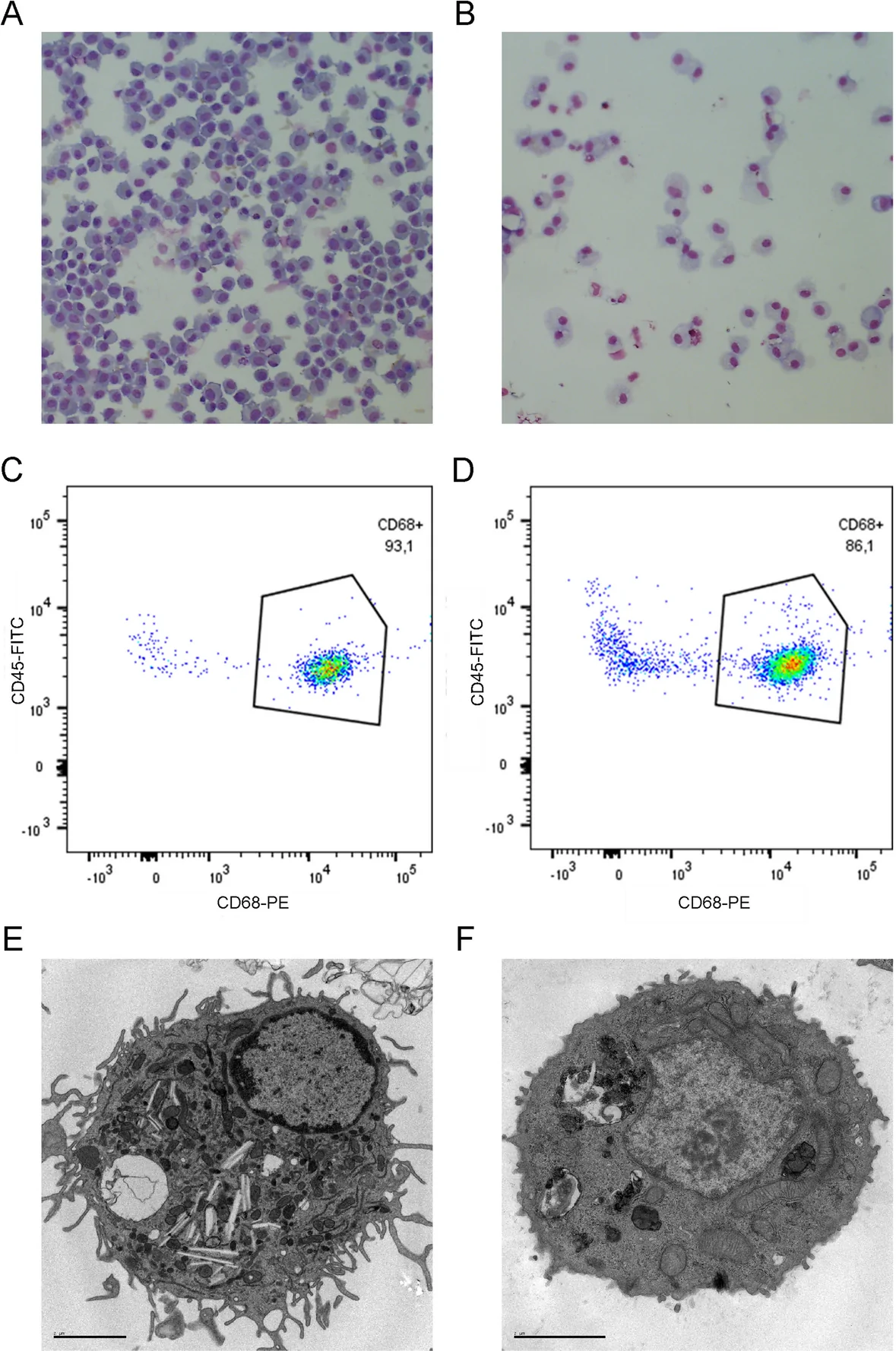
Characterization of AM and IM. **A** Diff-Quick stain of isolated AM (200 × magnification). **B** Diff-Quick stain of isolated IM (200 × magnification). **C** CD45 and CD68 staining in isolated AM evaluated by flow cytometry. Cells were immunostained with FITC-labelled anti-CD45 and PE-labelled anti-CD68 antibodies. **D** CD45 and CD68 staining in isolated IM evaluated by flow cytometry. Cells were immunostained with FITC-labelled anti-CD45 and PE-labelled anti-CD68 antibodies. **E** Transmission electron micrograph of an AM. **F** Transmission electron micrograph of an IM

Cell purity was assessed by Diff-Quick staining (Diagnostics Grifols S.A., Spain) on cytospin preparations in multiple independent experiments (*n* = 8), confirming 95.3 ± 1.3% AM and 84.9 ± 4.1% IM (Fig. 1A and B). Flow cytometry analyses (FACSCanto II, BD Pharmingen, USA) were performed using anti-CD45-FITC (Biolegend, Germany) and anti-CD68-PE antibodies (Acris, Germany). Appropriate isotype controls were included to define positive populations. Results showed 99.0 ± 1.4% CD45⁺ and 93.1 ± 3.6% CD68⁺ cells in AM preparations, and 86.1 ± 4.8% CD68⁺ cells in IM (Fig. 1C and D). Prior to antibody staining, cells were incubated with irrelevant IgG to block Fc receptors, minimizing non-specific binding. All assays were performed in triplicate, analysing 5 × 10^5^ cells per sample.

Transmission electron microscopy (TEM) was used to further characterize cell morphology. Cells were fixed in 2.5% glutaraldehyde in 0.1 M phosphate buffer (pH 7.4) for 2 h at 4 °C, post-fixed with 1% osmium tetroxide and 0.8% potassium ferrocyanide for 1 h at 4 °C, dehydrated through a graded acetone series (30% to 100%), embedded in Spurr resin, sectioned, stained with uranyl acetate and lead citrate, and imaged by a JEM 1010 electron microscope (Jeol Ltd., Tokyo, Japan) equipped with a Gatan/BioScan digital camera (Gatan Inc., Pleasanton, CA) (Fig. 1E and F).

#### Alveolar type II (ATII) cells

Fresh ATII cells were isolated from healthy donor rats using the same enzymatic digestion protocol up to the second DNase step. Cells were centrifuged at 250 × g for 20 min and resuspended in DCCM-1 medium supplemented with 2% L-glutamine. Differential adhesion was performed by plating cells for 2 h to minimize fibroblast contamination; non-adherent cells were then collected, washed twice with sterile saline, and resuspended for transplantation.

Cell viability was assessed by Trypan Blue exclusion, consistently exceeding 95%. Purity was evaluated by alkaline phosphatase staining of cytospin slides (Cytospin 3 centrifuge, Shandon Scientific Ltd., Grupo Taper, Spain), which identifies ATII cells by their high enzymatic activity; positivity averaged 85.9 ± 5.2% (*n* = 8). Additionally, flow cytometry for p180 lamellar body antigen (Abcam, UK), a marker specific for surfactant-producing lamellar bodies in ATII cells, showed 83.9 ± 4.8% positivity (*n* = 3). ATII cells were used fresh for transplantation or co-culture experiments. To assess preservation of the ATII phenotype under co-culture conditions, expression of ATII-associated surfactant markers and the ATI-associated marker aquaporin-5 was evaluated after 48 h of culture.

#### Fibroblasts

Fibroblasts were obtained from lung tissue explants of control and BLM-treated rats. Minced tissue (~ 1 mm^3^ pieces) was plated in RPMI-1640 supplemented medium and cultured. Outgrowth of fibroblasts was observed after 7 days, explants were removed before cells reached confluence (approximately 2 weeks). Cells were trypsinized and seeded at 40,000 cells/well in 24-well plates for co-culture experiments (passages 2–4). Fibroblast identity was initially confirmed morphologically and by adherence characteristics typical of mesenchymal cells. To further characterize the mesenchymal populations used in co-culture experiments, expression of myofibroblast-associated markers including α-SMA, Ccn2/CTGF, fibronectin, collagen I and collagen III was additionally assessed by immunostaining and RT-PCR.

#### Isolation and analysis of circulating CXCR4^+^ cells

Peripheral blood (1 mL) was processed to assess CXCR4 expression by RT-PCR. Peripheral blood mononuclear cells (PBMCs) were isolated from 5 mL of blood by density gradient centrifugation over Histopaque 1077 (Sigma-Aldrich) at 400 × g for 30 min. Mononuclear cells were washed and centrifuged on a 10% sucrose cushion at 250 × g for 10 min. Cells were cultured in DMEM supplemented with 20% fetal calf serum, and non-adherent cells were removed after 5 days. Adherent cells were maintained for 7–10 days, fixed, and immunostained for CXCR4. CXCR4^+^ cells were quantified in 15 randomized microscopic fields per sample using identical microscope settings and magnification for all experimental groups. Cell counts were independently assessed by two blinded observers. The total number of CXCR4^+^ cells per culture was estimated by extrapolating the average number of CXCR4^+^ cells per mm^2^ of field of view to the total surface area of the culture flask.

### In vivo studies

#### Bleomycin-induced lung fibrosis

Pulmonary fibrosis was induced by intratracheal administration of 2.5 U/kg BLM (Sigma-Aldrich, USA) dissolved in 400 µL sterile saline under isoflurane anesthesia. Control animals received sterile saline alone. Body weights and clinical signs were recorded every 2 days to monitor health status.

#### Transplantation procedure

At day 15 post-BLM instillation, corresponding to established fibrosis, animals received intratracheal transplantation of 2.5 × 10⁶ freshly isolated ATII cells suspended in 400 µL sterile saline. Control animals received ATII cells 15 days after saline instillation. At day 21 post-BLM instillation, animals were euthanized with an intraperitoneal overdose of sodium pentobarbital (200 mg/kg). Death was confirmed by the cessation of circulation. The lungs were then dissected, weighed and processed for downstream analyses.

#### Experimental groups

Animals were randomly assigned (*n* = 8 per group) to four groups after a one-week acclimatization period in the animal facility and before any experimental procedure. Animals were allocated to the four groups by randomly selecting individuals from the different home cages to ensure a balanced distribution across cages and to minimize potential cage-related effects. The four experimental groups were:

- C: Saline instillation (control).
- C + ATII: Saline instillation + ATII cell transplantation.
- BLM: Bleomycin instillation.
- BLM + ATII: Bleomycin instillation + ATII cell transplantation.

#### Sample size determination

The sample size (*n* = 8 rats per group) was determined a priori based on a power calculation performed using SigmaPlot 15.0 (Grafiti LLC, Palo Alto, CA). For the purpose of the sample size calculation, lung hydroxyproline content was selected as the primary outcome measure, as it provides a robust quantitative indicator of fibrosis severity in this model. Assuming a standard deviation of 1500 µg/lung, an expected difference of 3000 µg/lung between control and fibrotic groups, a two-tailed t-test, α = 0.05, and a statistical power of 0.95, the required sample size was calculated to be 8 animals per group. This sample size is consistent with our previous studies using the same model [23].

#### Animal welfare monitoring and analgesia

After BLM instillation, animals were monitored daily using a clinical supervision protocol adapted from the Morton and Griffiths framework and expanded for our pulmonary fibrosis model. Monitoring included body weight, general appearance, spontaneous behaviour, response to handling, and respiratory pattern, evaluated using a predefined scoring system. Scores of 0–4 indicate absent or mild distress and require routine daily monitoring. Scores of 5–9 reflect moderate to severe distress and, depending on the score, prompt intensified monitoring (twice daily) together with supportive measures, such as administering analgesia when indicated, placing food pellets inside the cage, and providing long-nozzle water bottles to facilitate access to food and water. A score of ≥ 10 is considered indicative of severe distress and constitutes a predefined humane endpoint in our protocol.

In this study, none of the animals reached the welfare score criteria requiring analgesic administration. Likewise, no animals reached the predefined humane endpoints during the study. If such endpoints had been met, euthanasia would have been carried out according to our established protocol for this model.

#### Inclusion and exclusion criteria

No predefined exclusion criteria were applied beyond the humane endpoints described above. All animals completed the study and were included in the analyses.

#### Hydroxyproline assay

Lung collagen content was quantified by measuring hydroxyproline using Woessner's method. Lung tissue samples were hydrolyzed with 6 M HCl for 18 h at 110 °C, neutralized, and reacted sequentially with chloramine-T, perchloric acid, and dimethylaminobenzaldehyde. Absorbance was measured at 550 nm. Hydroxyproline concentration was calculated based on a hydroxyproline standard curve and expressed as μg/lung.

#### Histology and immunostaining

Left lungs were perfusion-fixed with 10% neutral buffered formalin at a constant pressure of 25 cm H₂O for 12–24 h, embedded in paraffin, and sectioned at 4 µm. Sections were stained with Masson's trichrome to evaluate connective tissue and collagen deposition.

For immunohistochemistry, antigen retrieval was performed by heating sections in sodium citrate buffer (pH 6.0) at 90 °C for 30 min. Endogenous peroxidase activity was blocked with 5% hydrogen peroxide for 30 min. Non-specific binding was blocked by incubating sections with 3% fetal calf serum and 1% bovine serum albumin for 2 h at room temperature. Primary antibodies against CD68, CXCR4, and CXCL12 were applied overnight at 4 °C (1:200 dilution). Sections were incubated sequentially with biotinylated secondary antibodies and streptavidin-HRP (both 1:200, 1 h each). Diaminobenzidine (DAB) was used as chromogen, and sections counterstained with hematoxylin. Omission of primary antibody served as negative control. Immunostained macrophages were counted in 15 random fields per section by two independent observers blinded to group allocation.

### In vitro co-culture studies

In order to determine whether the phenotypic modulation of macrophages observed in vivo was directly mediated by ATII cells, we designed co-culture experiments between macrophages and ATII cells under defined conditions.

#### Macrophage-ATII cells co-culture

**Fig. 2:**
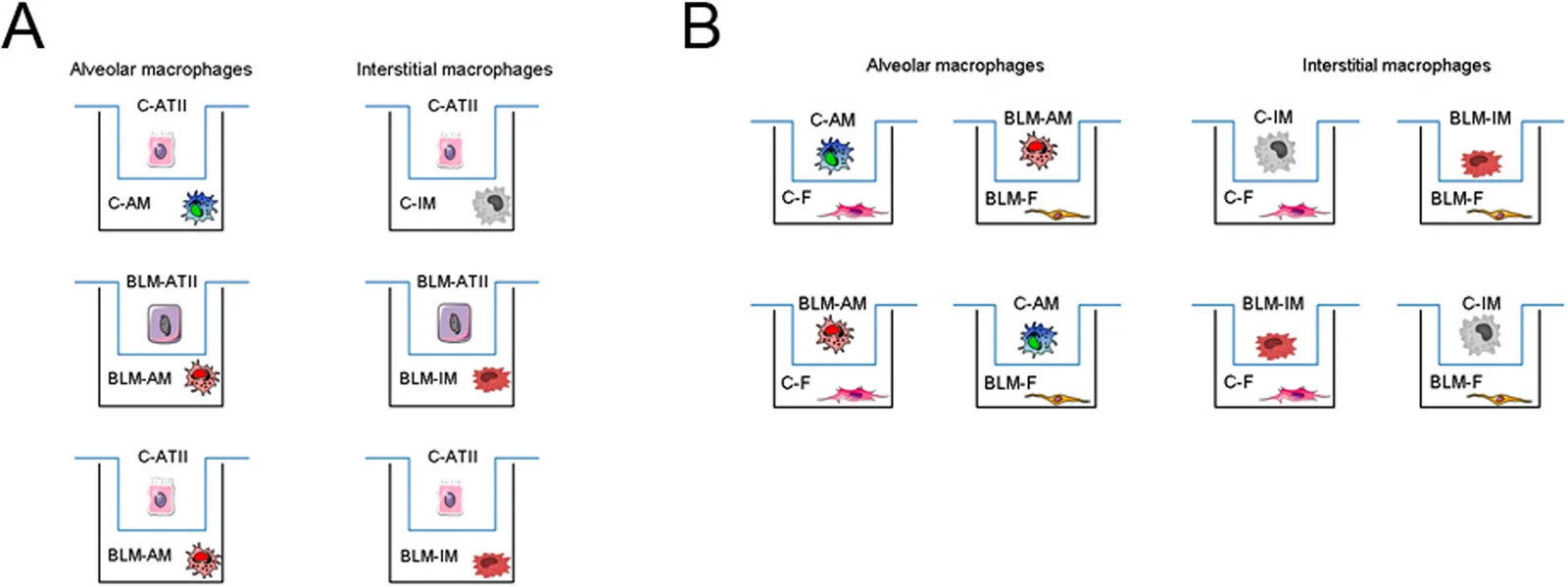
Scheme of co-culture experiments. **A** Scheme of co-cultures between pulmonary macrophages and ATII cells. **B** Scheme of co-cultures between pulmonary macrophages and fibroblasts

AM or IM, isolated from control (C-AM, C-IM) or BLM-treated (BLM-AM, BLM-IM) lungs, were seeded at 35,000 cells/mL in 24-well plates and allowed to adhere for 1 h. ATII cells from control or BLM animals (C-ATII, BLM-ATII) were seeded at the same density on transwell inserts with 0.4 µm pore size (Corning VWR International Ltd., Ewloe, Flintshire, UK), allowing exchange of soluble factors but preventing direct cell-to-cell contact, and co-cultured for 48 h (Fig. 2A). Cultures were maintained in RPMI-1640 supplemented with 10% fetal bovine serum, 1% penicillin–streptomycin, 1% *Mycokill*, and 1% Amphotericin B. After co-culture, cells were harvested separately for RNA extraction using TRIzol® (Invitrogen, Life Technologies, Spain).

To determine how distinct macrophage activation states, influence fibroblast responses, we also established co-culture systems under defined conditions.

#### Macrophage-fibroblast co-culture

Fibroblasts from control (C-F) or BLM (BLM-F) lungs were seeded at 35,000 cells/mL in 24-well plates and allowed to adhere for 1 h. AM or IM from control or BLM lungs were seeded at equal density onto 0.4 µm transwell inserts and co-cultured with fibroblasts for 48 h (Fig. 2B). Subsequently, both cell types were separately harvested for RNA isolation and assessment of fibrotic markers, including TGF-β, α-SMA, collagen type I, and collagen type III via real-time PCR.

#### RNA isolation and real-time PCR

Total RNA was extracted with TRIzol reagent, reverse-transcribed, and amplified using SYBR Green chemistry on a Bio-Rad DNA Engine qPCR system. Gene-specific primers (listed in Table 1) were used. Expression levels were normalised to GAPDH as housekeeping gene. Both technical triplicates and biological replicates (*n* = 4) were analysed. Relative gene expression was calculated using the ΔΔCt method and expressed as fold-change over control groups.

**Table 1:** Rat primers used in RT-PCR analysis

Primers	Forward	Reverse
TNF-α	5’-AACTCCCAGAAAAGCAAGCA-3’	5’-CGAGCAGGAATGAGAAGAGG-3’
IL1-β	5’-AAAAATGCCTCGTGCTGTCT-3’	5’-TCGTTGCTTGTCTCTCCTTG-3’
Mannose Receptor	5’-GCAGGTGGTTTATGGGATGT-3’	5’-GGGTTCAGGAGTTGTTGTGG-3’
Arginase-I	5’-GGGAAGACACCAGAGGAGGT-3’	5’-TGATGCCCCAGATGACTTTT-3’
IL-10	5’-CATCCGGGGTGACAATAA-3’	5’-TGTCCAGCTGGTCCTTCT-3’
TGF-β	5’- TGAGTGGCTGTCTTTTGACG-3’	5’- TGGGACTGATCCCATTGATT-3’
α-SMA	5’- TGTGCTGGACTCTGGAGATG-3’	5’- GAAGGAATAGCCACGCTCAG-3’
Collagen I	5’- AATGGTGCTCCTGGTATTGC-3’	5’- GGTTCACCACTGTTGCCTTT-3’
Collagen III	5’- GTCCACGAGGTGACAAAGGT-3’	5’- CATCTTTTCCAGGAGGTCCA-3’
CXCR4	5’- CGGTCATCCTTATCCTGGCT −3’	5’- GAGATCCACTTGTGCACGAC −3’
CXCL12	5’- CGTTCCTGATCTCTGCTTGC −3’	5’- TCTCCTTCCTTCCACTG −3’
GAPDH	5’-CTGTGTCTTTCCGCTGTTTTC-3’	5’-TGTGCTGTGCTTATGGTCTCA-3’

#### Statistical analysis

Data are presented as mean ± standard deviation (SD). Individual data points were overlaid in all bar graphs to improve visualization of biological variability and data distribution. Statistical significance was determined by one-way ANOVA followed by Tukey’s multiple comparisons post hoc test (GraphPad Software Inc., La Jolla, CA). Differences with *p* < 0.05 were considered statistically significant. For all in vivo experiments, each rat was considered the experimental unit. For in vitro co-culture studies, each independent cell preparation obtained from a different donor animal was treated as one biological replicate. Technical replicates were averaged prior to analysis.

## Results

### In vivo BLM model and effects of ATII cell transplantation on macrophage dynamics

**Fig. 3:**
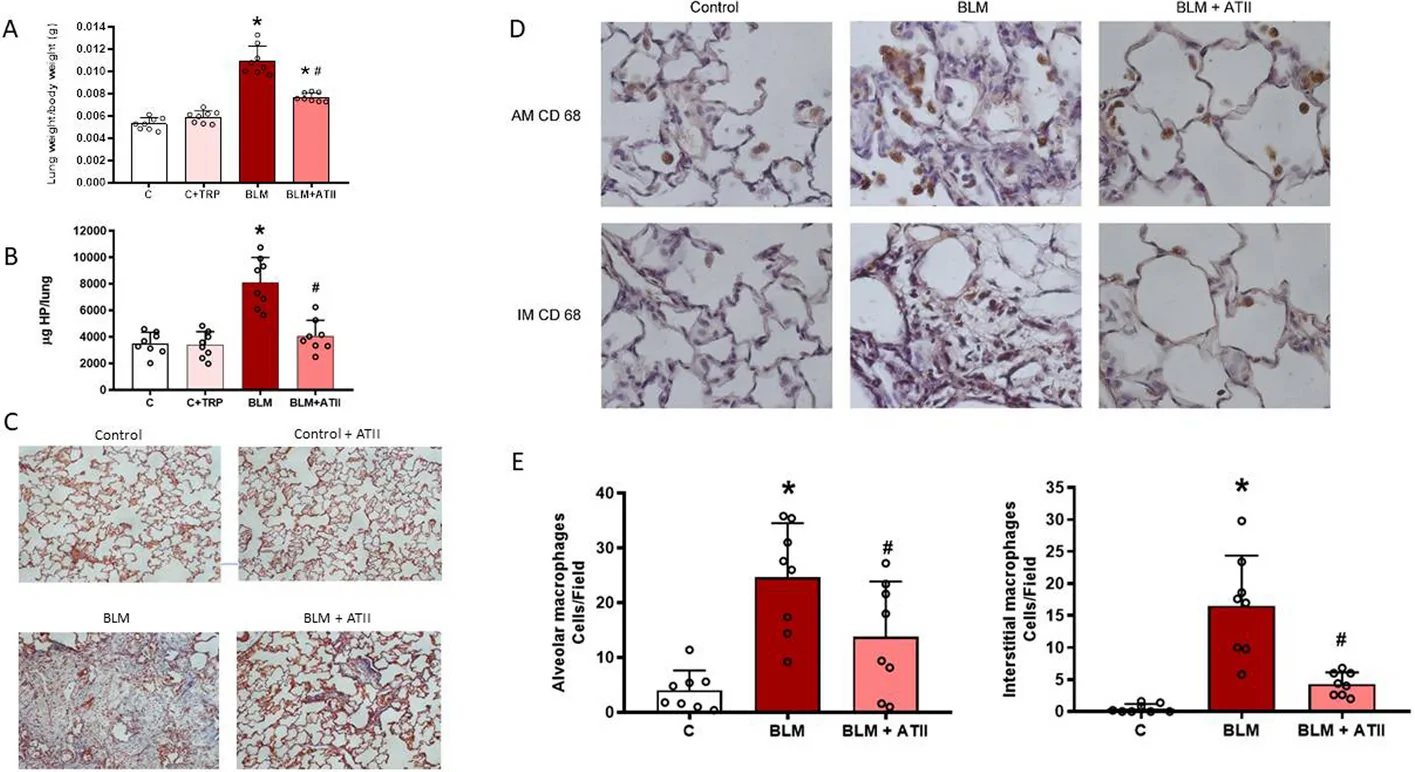
In vivo BLM model and effects of ATII cell transplantation on macrophage dynamics. **A** Lung weight-to-body weight ratio after 21 days of BLM-induced lung fibrosis. **B** Lung hydroxyproline content after 21 days of BLM-induced pulmonary fibrosis. **C** Representative bright-field photomicrographs of lung sections stained with Masson’s trichrome after 21 days of BLM-induced pulmonary fibrosis (400 × magnification). **D** Immunohistochemical staining of pulmonary macrophages using anti-CD68 antibody. Upper panels show BAL-space CD68⁺ macrophage-enriched populations operationally referred to as alveolar macrophages (AM); lower panels show interstitial/fibrotic-area CD68⁺ macrophage-enriched populations operationally referred to as interstitial macrophages (IM) (400 × magnification). **E** Quantification of CD68⁺ macrophages in AM (left) and IM (right) compartments. Data are expressed as mean ± SD (*n* = 8 per group). *p < 0.05 vs. control; #*p* < 0.05 vs. BLM

BLM instillation induced an increase in lung weight relative to total body weight, which was partially reduced in the BLM + ATII cell transplanted group (Fig. 3A). Lung hydroxyproline levels, a marker of collagen deposition, were significantly increased after BLM instillation. In contrast, ATII cell transplantation significantly reduced hydroxyproline levels to control values (Fig. 3B). These results were confirmed by Masson trichrome staining of tissue sections (Fig. 3C). Lungs from the BLM group showed marked peribronchiolar and interstitial infiltration of inflammatory cells, extensive thickening of the intralveolar septa, oedema, increased accumulation of fibroblast-like interstitial cells, and excess collagen deposition. Conversely, although multifocal parenchymal lesions were still present in the BLM + ATII cell transplanted group, fibrotic areas were smaller and considerably fewer than those observed in the non-transplanted BLM group. Moreover, large regions of preserved alveolar architecture were evident, indicating marked attenuation of fibrosis.

Fibrotic lungs exhibited a marked expansion of CD68⁺ cells in both alveolar spaces (AM) and the pulmonary interstitium, including within fibrotic foci (IM) (Fig. 3D). Notably, following ATII cell transplantation, the number of macrophages infiltrating both compartments was significantly reduced (*p* < 0.05 for AM and *p* < 0.01 for IM) (Fig. 3E).

### Effect of ATII transplantation on macrophage activation profiles

**Fig. 4:**
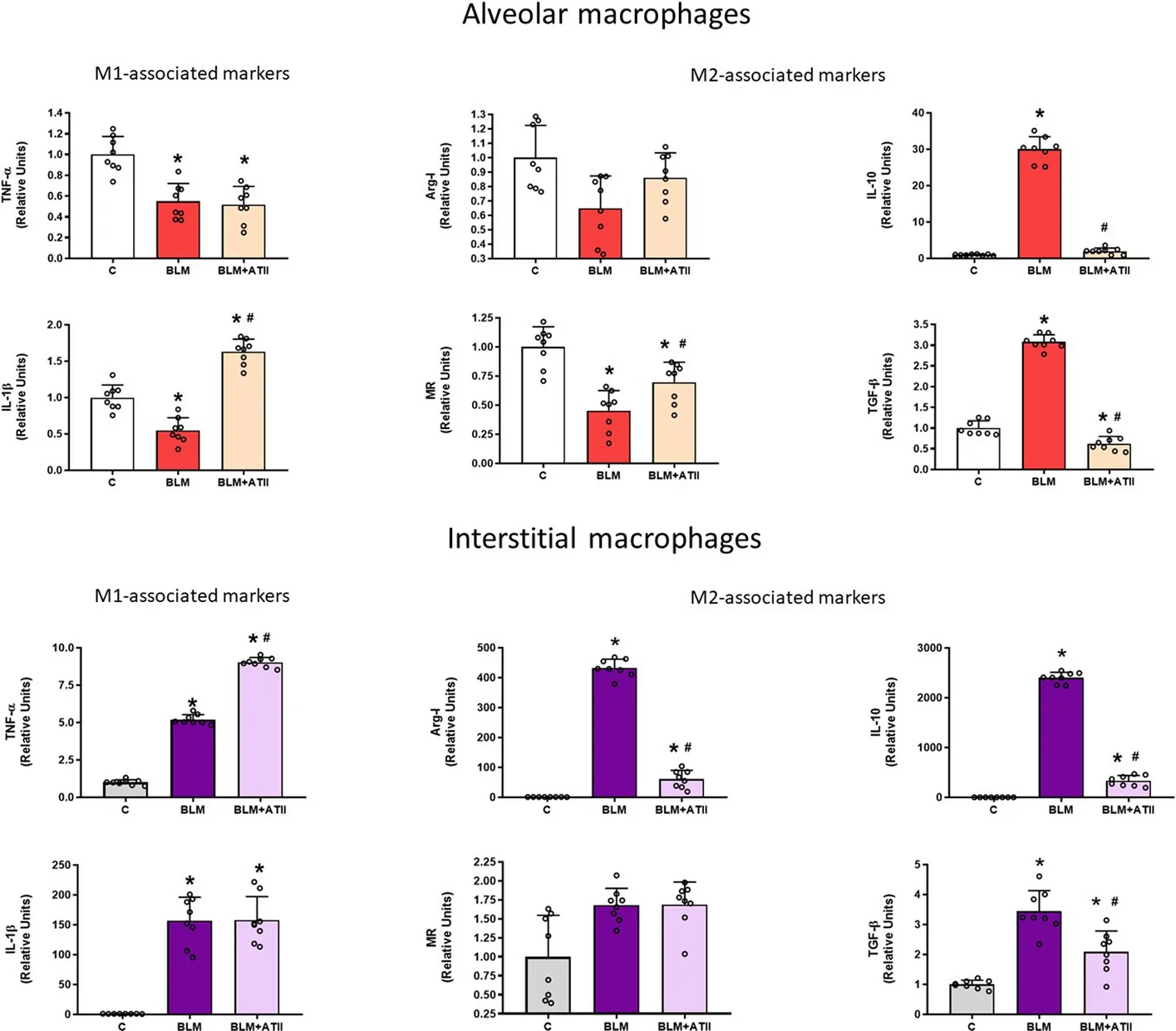
Effect of ATII transplantation on macrophage activation profiles. mRNA expression of M1-associated markers (TNF-α, IL-1β) and M2-associated markers (MR, Arg-1, IL-10, TGF-β) in AM and IM. Data are expressed as mean ± SD (*n* = 8). *p < 0.05 vs. control; #*p* < 0.05 vs. BLM

To evaluate activation states of AM and IM, the expression levels of M1-associated markers (TNF-α, IL-1β) and M2-associated markers (Arg-1, MR, IL-10, TGF-β) were determined (Fig. 4).

In AM (Fig. 4A), expression of M1-associated markers (TNF-α, IL-1β) was significantly reduced (*p* < 0.05) in the BLM group compared with controls. Among M2-associated markers, IL-10 and TGF-β were significantly increased (*p* < 0.001), whereas MR expression was significantly reduced and Arg-1 remained unchanged. Following ATII cell transplantation, IL-10 and TGF-β expression were significantly reduced to control levels, MR expression was partially restored toward control values, and IL-1β expression significantly increased, whereas TNF-α levels were not restored. These results indicate that AM acquire a profibrotic activation profile enriched in selected M2-associated markers during fibrosis, and that ATII cell transplantation partially reverses this activation profile, primarily through reduction of IL-10 and TGF-β expression.

By contrast, in IM (Fig. 4B), a mixed activation profile was observed, with increased expression of both M1-associated and M2-associated markers, consistent with the heterogeneous macrophage states described in single-cell transcriptomic studies [14, 19]. In the BLM group, M1-associated markers were significantly increased. ATII transplantation did not reverse this increase; indeed, TNF-α expression was further elevated in the BLM + ATII group. Among M2-associated markers, Arg-1, IL-10, and TGF-β were significantly increased in the BLM group compared with controls. ATII cell transplantation significantly reduced Arg-1, IL-10, and TGF-β expression, although not all markers returned fully to control levels. These findings suggest that ATII cell transplantation partially attenuates the profibrotic M2-associated activation profile in IM while maintaining, and in some cases enhancing, expression of pro-inflammatory markers.

### ATII epithelial marker expression during co-culture conditions

To determine whether freshly isolated ATII cells retained key epithelial features during the 48 h co-culture period, expression of surfactant protein C (SP-C), a specific ATII marker, surfactant proteins SP-A, SP-B and SP-D, and the ATI-associated marker aquaporin-5 (Aqp-5) was analysed (Supplementary Fig. S1). After 48 h of co-culture with control AM or IM, surfactant-related gene expression showed a general downward trend, with significant reductions in SP-A and SP-D in ATII cells co-cultured with control AM. A similar trend was observed when ATII cells were co-cultured with macrophages from BLM-treated animals, with significant reductions in SP-B in the BLM-AM condition and in SP-D in both BLM-AM and BLM-IM conditions. Importantly, Aqp-5 expression did not increase above levels detected in freshly isolated ATII cells in any condition and was significantly reduced in ATII cells co-cultured with control AM, BLM-AM and BLM-IM. These findings suggest that ATII cells undergo partial adaptation to ex vivo co-culture conditions, reflected by reduced surfactant-related gene expression, but do not show evidence of early transition toward an ATI-like phenotype during the 48 h experimental window.

### Co-culture assays to dissect macrophage-ATII crosstalk

**Fig. 5:**
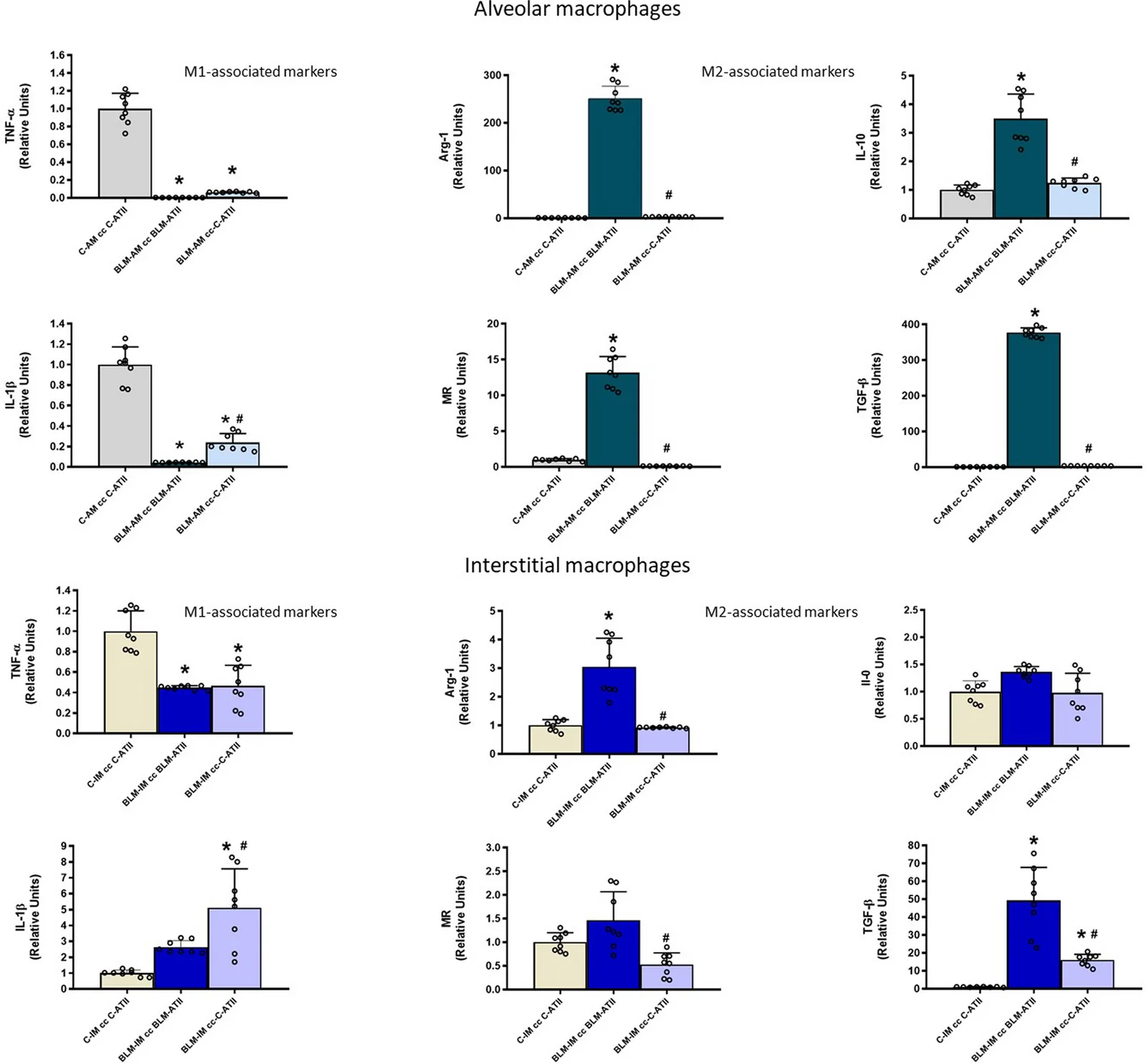
Co-culture assays to dissect macrophage-ATII crosstalk. mRNA expression of M1-associated markers (TNF-α, IL-1β) and M2-associated markers (MR, Arg-1, IL-10, TGF-β) was analyzed in three co-culture conditions designed to mimic the in vivo experimental conditions: control-AM with control-ATII (control), BLM-AM with BLM-ATII (fibrotic), and BLM-AM with control-ATII (transplant mimic). Co-cultures were performed for both AM (**A**) and IM (**B**) using transwell inserts (0.4 µm pores) to allow exchange of soluble factors without direct cell contact. Data are expressed as mean ± SD (*n* = 8). *p < 0.05 vs. control; #*p* < 0.05 vs. BLM

Based on our in vivo findings that ATII cell transplantation modified macrophage profiles, we designed co-culture experiments to further examine macrophage-ATII cell crosstalk under specific controlled conditions (Fig. 5). Co-cultures were established between control-AM and control-ATII cells (mimicking the control condition), BLM-AM and BLM-ATII cells (mimicking the fibrotic condition), and BLM-AM and control-ATII cells (transplant mimic). The same design was applied to the IM population.

In AM (Fig. 5A), BLM-AM co-cultured with BLM-ATII showed a significant decrease (*p* < 0.001) in M1-associated markers (TNF-α, IL-1β) and a significant increase (*p* < 0.001) in all M2-associated markers (MR, Arg-1, IL-10, TGF-β) compared to controls. When BLM-AM were co-cultured with C-ATII, M2-associated markers were significantly reduced to control levels, whereas M1-associated marker expression showed a recovery trend.

In IM (Fig. 5B), BLM-IM co-cultured with BLM-ATII exhibited significantly reduced TNF-α expression and significantly increased Arg-1 and TGF-β levels, while IL-1β, MR, and IL-10 did not change significantly compared to controls. When BLM-IM were co-cultured with C-ATII, IL-1β expression significantly increased, whereas Arg-1 and TGF-β expression were significantly reduced. However, TGF-β levels did not fully return to control values.

Altogether, our in vivo results show that ATII cell transplantation shifts AM and IM toward a less profibrotic profile, likely through direct and indirect intercellular signalling. In vitro studies confirmed that part of this modulatory effect occurs via soluble factors, as macrophage activation profiles were altered despite the absence of direct cell-cell contact with ATII cells.

### Fibroblast activation modulated by macrophage activation states

**Fig. 6:**
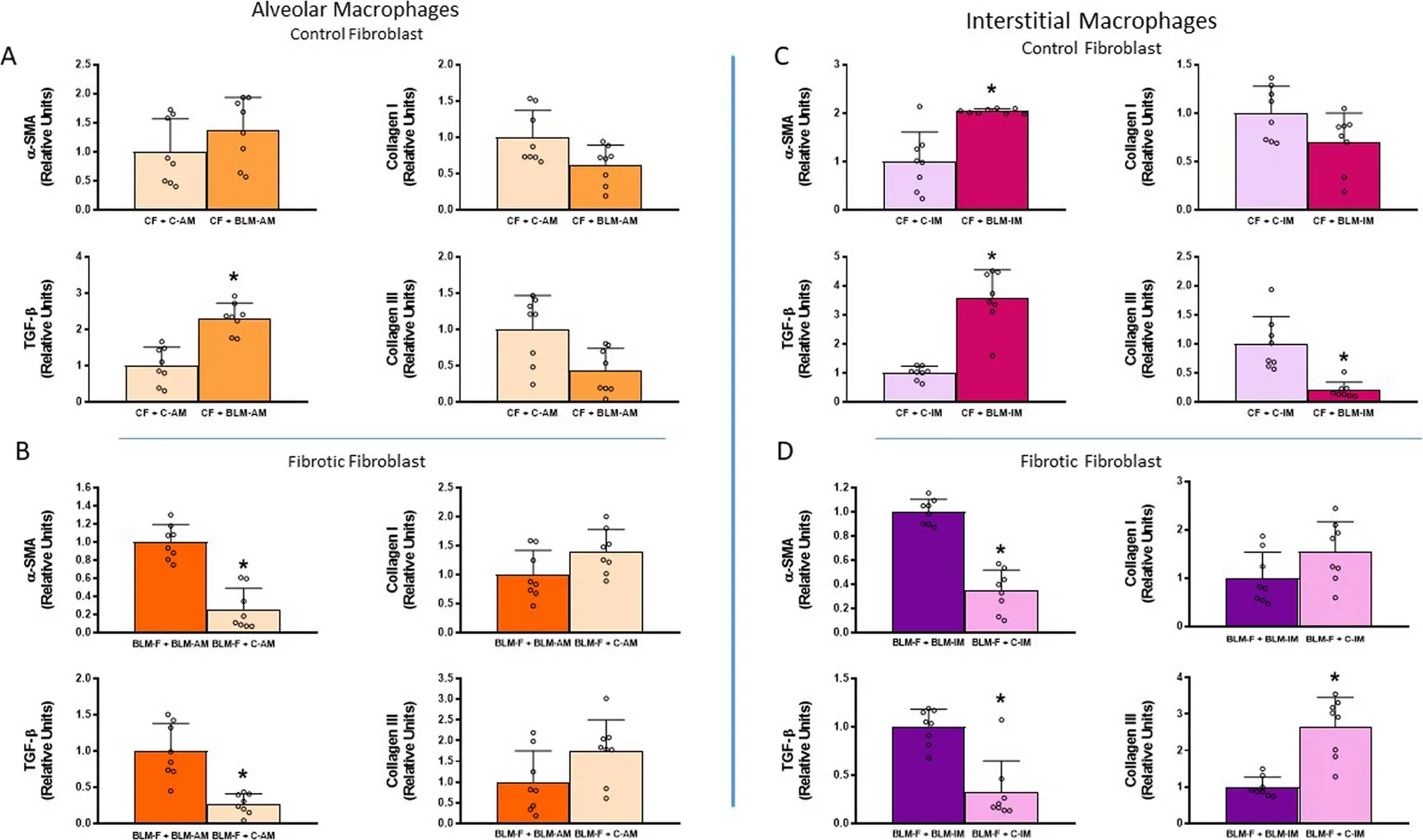
Fibroblast activation modulated by macrophage activation states. mRNA expression of fibroblast activation markers (α-SMA, TGF-β, Collagen I, Collagen III). **A** AM from control or BLM animals co-cultured with fibroblasts from control animals. **B** AM from control or BLM animals co-cultured with fibroblasts from fibrotic animals. **C** IM from control or BLM animals co-cultured with fibroblasts from control animals. **D** IM from control or BLM animals co-cultured with fibroblasts from fibrotic animals. Data are expressed as mean ± SD (*n* = 8). *p < 0.05 vs. control; #*p* < 0.05 vs. BLM

After observing the ability of ATII cells after transplantation to modulate macrophage profiles, we performed a series of co-culture experiments to assess whether different macrophage activation states influence fibroblast behaviour. For this, we used macrophages isolated from two distinct experimental conditions: control animals, in which macrophages exhibit a basal phenotype, and BLM-treated animals, where macrophages display a profibrotic activation profile enriched in M2-associated markers, as previously demonstrated in our model. The mesenchymal phenotype of explant-derived fibroblast cultures was further confirmed by expression of canonical myofibroblast-associated markers (Supplementary Fig. S2). These macrophages were co-cultured with fibroblasts also isolated either from control or fibrotic lungs (Fig. 6). Transwell inserts were again used to permit soluble mediator exchange without direct cell–cell contact. The experimental combinations were as follows: C-F (control-fibroblast) with C-AM (control-AM) or with BLM-AM (fibrotic-AM); BLM-F (fibrotic-fibroblast) with BLM-AM (fibrotic-AM) or with C-AM; C-F with C-IM (Control-IM) or with BLM-IM (fibrotic-IM); and BLM-F with BLM-IM or with C-IM.

In C-F co-cultured with BLM-AM, the most relevant finding was a significant induction of the expression of TGF-β, a key fibrogenic cytokine, while α-SMA, collagen I, and collagen III gene expression remained unchanged (Fig. 6A). This result suggests that fibrotic AM may induce some fibrogenic signalling in fibroblasts, although it does not lead to full fibroblast activation.

In contrast, BLM-F co-cultured with C-AM showed a significant reduction in α-SMA and TGF-β expression, whereas collagen I and collagen III expression was not significantly modified (Fig. 6B), suggesting the paracrine modulatory role of AM on fibroblast activation under fibrotic conditions.

When C-F were co-cultured with BLM-IM, α-SMA and TGF-β expression were significantly increased, and collagen III was significantly reduced, while collagen I remained unchanged (Fig. 6C). These results suggest that fibrotic IM promote fibroblast activation and may alter extracellular matrix composition.

Notably, C-IM co-cultured with BLM-F reduced α-SMA and TGF-β expression and increased collagen III levels, without affecting collagen I (Fig. 6D). This indicates that control IM can attenuate fibroblast activation and help restore extracellular matrix homeostasis.

In summary, macrophage activation status exerted a differential influence on fibroblast activation: fibrotic macrophages isolated from BLM animals promoted partial fibrogenic activation of control fibroblasts, while basal macrophages isolated from control animals attenuated the activation of fibrotic fibroblasts, including changes in collagen expression.

### CXCL12/CXCR4 axis and circulating CXCR4^+^ cells

**Fig. 7:**
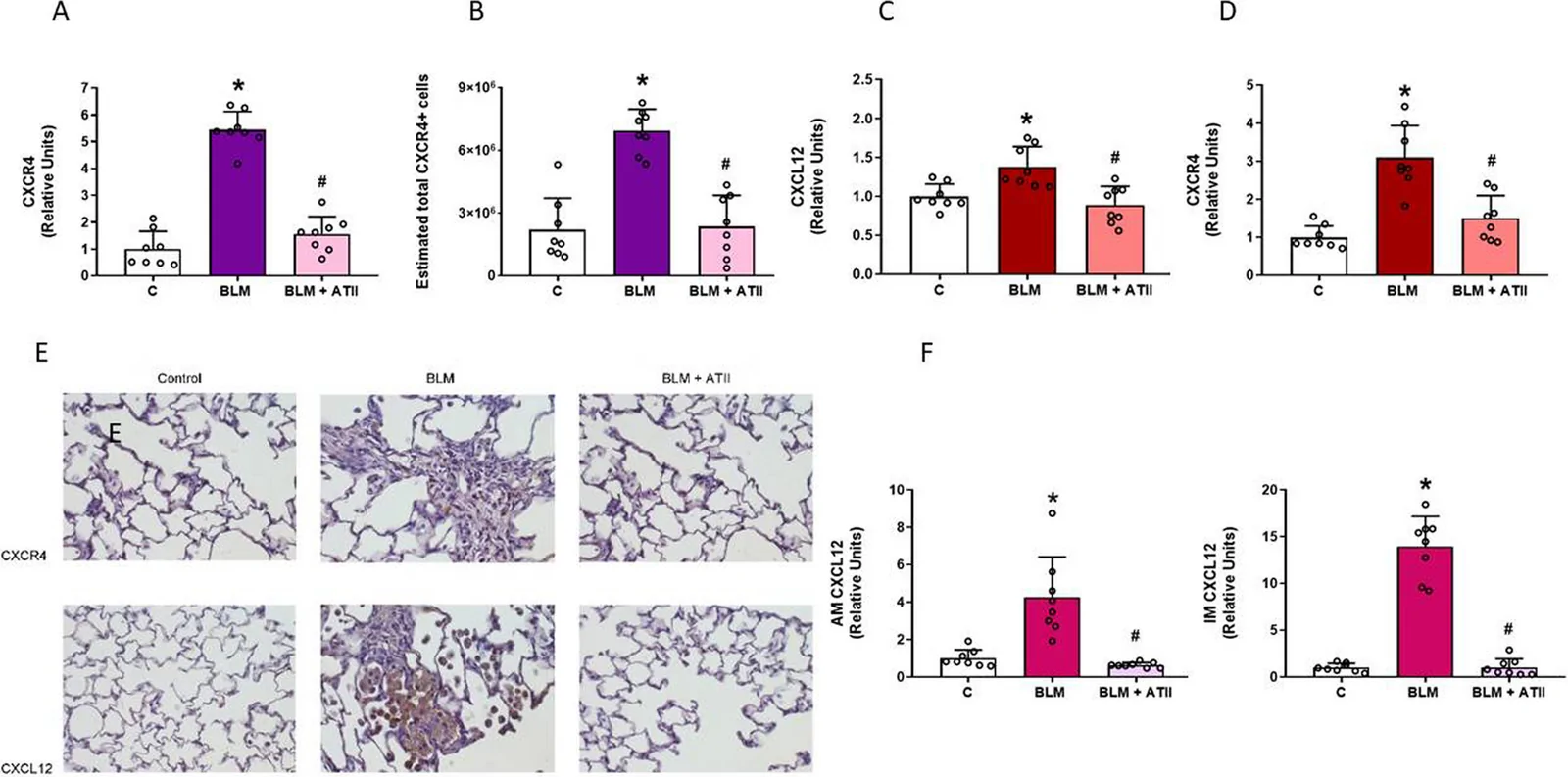
CXCL12/CXCR4 axis. **A** mRNA expression of CXCR4 in blood. **B** Number of circulating CXCR4^+^ cells in blood. **C** CXCL12 expression in lung tissue. **D** CXCR4 expression in lung tissue. **E** Immunohistological staining with anti-CXCR4 and anti-CXCL12 in lung tissue Sects. (400 × magnification). **F** CXCL12 mRNA expression in isolated AM. **G** CXCL12 mRNA expression in isolated IM. Data are expressed as mean ± SD (*n* = 8). *p < 0.05 vs. control; #*p* < 0.05 vs. BLM

Given the observed changes in macrophage activation induced by ATII cell transplantation, we next investigated whether this therapy could modulate the CXCL12/CXCR4 axis and the accumulation of circulating CXCR4^+^ cells (Fig. 7). The CXCL12/CXCR4 axis is a key pathway involved in the recruitment of circulating CXCR4^+^ cells and has previously been implicated in fibrotic processes. We therefore evaluated whether modulation of macrophage activation by ATII cell transplantation was associated with changes in this signalling axis and in the number of circulating CXCR4^+^ cells. Fibrotic animals showed a significant increase in CXCR4 mRNA expression in total peripheral blood (Fig. 7A), as well as an increased number of circulating CXCR4^+^ cells (Fig. 7B), compared to controls. Following ATII cell transplantation, both parameters were significantly reduced. These findings suggest that ATII cell therapy reduces the accumulation of circulating CXCR4^+^ cells during pulmonary fibrosis. To further explore the CXCL12/CXCR4 axis in the lung, we analysed the expression of both chemokines in fibrotic tissue. CXCL12 (Fig. 7C) and CXCR4 (Fig. 7D) expression were significantly increased in fibrotic lungs (BLM), whereas ATII cell transplantation restored their levels to baseline. These findings were confirmed by immunostaining, which revealed CXCR4 primarily localized in fibrotic areas, while CXCL12 expression was mainly observed in macrophages (Fig. 7E).

To further define the macrophage contribution, CXCL12 expression was analysed in isolated AM and IM from each group (Fig. 7F, G). CXCL12 was markedly upregulated in both macrophage populations during fibrosis, with a greater increase in IM. These increases were restored toward control levels.

Overall, ATII cell transplantation normalised CXCL12 expression in pulmonary macrophages and reduced the number of circulating CXCR4^+^ cells, suggesting that modulation of the CXCL12/CXCR4 axis may contribute to limiting the accumulation of circulating CXCR4^+^ cells during fibrosis.

## Discussion

Intratracheal transplantation of ATII cells has been shown to reverse established pulmonary fibrosis through multiple mechanisms [22–26, 30, 31]. In this study, we demonstrate that these benefits extend beyond epithelial repair, involving robust immunomodulation of macrophages and disruption of profibrotic cell-cell communication within the lung. Our data reveal that ATII cell therapy reduces both AM and IM accumulation and is associated with a shift from a profibrotic activation profile enriched in M2-associated markers toward a less profibrotic, more homeostatic activation profile. This reprogramming occurred without inducing classical M1 inflammation, suggesting restoration of immune equilibrium rather than activation of a proinflammatory response.

These findings are particularly relevant considering that most studies addressing macrophage modulation in pulmonary fibrosis have focused on early inflammatory or fibrogenic stages, primarily aiming to prevent rather than reverse disease progression [13, 32–34]. In contrast, our results demonstrate that macrophage reprogramming can also occur when fibrosis is already established and inflammation has subsided, specifically through the actions of transplanted ATII cells. This highlights the therapeutic adaptability of the fibrotic lung microenvironment and supports the feasibility of macrophage-targeted interventions driven by epithelial restoration at advanced disease stages.

Accordingly, the M1/M2 terminology used throughout this study should be interpreted as a simplified functional framework based on selected marker expression patterns rather than as evidence of discrete macrophage subsets. This is particularly relevant in fibrotic lungs, where macrophage populations exhibit marked heterogeneity and may include resident and monocyte-derived transitional states. Macrophage heterogeneity and plasticity are well-established features of pulmonary fibrosis, with IM exerting stronger profibrotic influence due to their proximity to fibroblasts and hybrid M1/M2 activation profile [13, 18, 35]. Our results are consistent with previous studies showing that both AM and IM acquire profibrotic characteristics during fibrosis, although IM display higher expression of IL-10 and Arg-1, both linked to fibroblast activation and matrix deposition [13, 35–39]. These findings align with recent single-cell analyses demonstrating that monocyte-derived macrophages sustain a spatially restricted fibrotic niche [33]. Our in vitro co-culture experiments further support this notion, showing that healthy ATII cells can partially modulate profibrotic macrophage activation profiles through soluble mediators, reinforcing their capacity to influence macrophage activation states. The ability of ATII cells to modulate this macrophage compartment underscores a novel layer of immune regulation driven by epithelial-macrophage interactions.

The patterns of macrophage activation observed are compatible with distinct effects on fibroblast behaviour, as previously described. Fibrotic AM and IM promoted fibroblast activation, whereas macrophages from control lungs exerted antifibrotic effects, consistent with their context-dependent plasticity [13, 17, 40]. By rebalancing macrophage activation through ATII cell transplantation, we likely disrupt profibrotic signalling cascades and feedback loops between macrophages and fibroblasts, thereby contributing to matrix remodelling and fibrosis resolution. Comparable modulation of macrophage function through direct targeting has been shown to alleviate fibrosis [34], and our data extend these findings by demonstrating that paracrine signals released by healthy ATII cells can promote macrophage reprogramming within the fibrotic lung.

We acknowledge that explant-derived cultures likely contain heterogeneous mesenchymal populations, including activated fibroblasts/myofibroblasts. However, the expression of canonical profibrotic markers supports the relevance of these cultures for modelling fibroblast activation within the fibrotic microenvironment.

In addition to local immune modulation, ATII therapy normalised the CXCL12/CXCR4 chemokine axis, reducing the number of circulating CXCR4⁺ cells, and their presence within fibrotic areas [41, 42]. This pathway links macrophage activation to the recruitment of circulating CXCR4⁺ cells, a heterogeneous population previously implicated in stromal expansion. Such mechanisms may contribute to the systemic antifibrotic effects of ATII cell transplantation. While fibroblasts, endothelial cells, and vascular smooth muscle cells are recognised as the primary sources of CXCL12 in lung tissue [21, 34], activated macrophages in the fibrotic microenvironment may also contribute to local CXCL12 production, thereby amplifying profibrotic signalling [10]. Together, these findings support a complex mechanism largely mediated by macrophage modulation; however, direct effects of ATII cells on CXCR4⁺ cell mobilisation and CXCL12 production warrant further investigation.

Our findings also highlight that macrophage modulation can only be sustained within a restored microenvironment. Previous work from our group demonstrated that transplantation of healthy macrophages alone, once fibrosis was established, was not sufficient to reverse the disease, indicating that the persistent fibrotic milieu can override their reparative properties [22]. In contrast, ATII cell transplantation remodels the epithelial-stromal interface, providing a reparative context that supports long-term macrophage reprogramming and tissue recovery.

This study has several limitations. First, macrophage populations were operationally defined according to BAL-derived and post-BAL lung tissue-derived enrichment procedures and were not resolved using lineage-specific or single-cell approaches. Therefore, these populations likely include heterogeneous resident and monocyte-derived macrophage subsets, particularly under fibrotic conditions. Second, the circulating CXCR4⁺ cells characterised in this study were identified solely on the basis of adherence selection and CXCR4 expression. As noted in the Introduction, CXCR4⁺ cells, including fibrocytes, have been implicated in fibrogenesis in previous reports; however, CXCR4 is not specific to fibrocytes and is also expressed by several other immune and hematopoietic cell populations. Our findings should therefore be interpreted as reflecting changes in a heterogeneous circulating CXCR4⁺ cell population, without implying a fibrocyte-specific identity. Third, although ATII cells showed reduced surfactant-related gene expression after 48 h under co-culture conditions, aquaporin-5 was not induced, suggesting no clear transition toward an ATI-like phenotype during the experimental window. Nevertheless, dynamic epithelial adaptation to ex vivo culture conditions cannot be fully excluded. Finally, the M1/M2 framework used in this study represents a simplified functional approximation of macrophage activation states within the complex fibrotic microenvironment.

In conclusion, our study identifies macrophage reprogramming as one of the mechanisms by which ATII cell transplantation promotes fibrosis resolution. By integrating epithelial regeneration with immune and stromal modulation, ATII cell therapy emerges as a comprehensive approach capable of restoring tissue homeostasis and offering disease-modifying potential for IPF and related disorders.

## Supplementary Information

Supplementary Material 1.

## Data Availability

The data presented in this study are available upon request to the corresponding author.

## Abbreviations

Arg-1: Arginase-1
ATII: Alveolar type II cells
BLM: Bleomycin
C: Control group
CXCL12: C-X-C motif chemokine ligand 12
CXCR4: C-X-C chemokine receptor type 4
DAB: 3,3'-Diaminobenzidine
DMEM: Dulbecco’s Modified Eagle Medium
ECM: Extracellular matrix
FBS: Fetal bovine serum
GAPDH: Glyceraldehyde-3-phosphate dehydrogenase
HCl: Hydrochloric acid
HRP: Horseradish peroxidase
IL-1β: Interleukin-1 beta
IL-10: Interleukin-10
IM: Interstitial macrophages
iPSC: Induced pluripotent stem cells
MR: Mannose receptor
PBS: Phosphate buffered saline
PCR: Polymerase chain reaction
qPCR: Quantitative polymerase chain reaction
RPMI: Roswell Park Memorial Institute medium
SD: Standard deviation
SMA (α-SMA): Alpha-smooth muscle actin
TGF-β: Transforming growth factor-beta
TNF-α: Tumour necrosis factor-alpha
