# Supplementary material for: Alveolar type II cell therapy ameliorates pulmonary fibrosis by reprogramming macrophage activation
Source: Inflamm Regen. 2026 Jul 10;46:38. doi: 10.1186/s41232-026-00434-4 (PMC13647792; doi:10.1186/s41232-026-00434-4)
Supplement: Supplementary file 1 [file 41232_2026_434_MOESM1_ESM.docx]

**Supplementary Material**

**Alveolar Type II Cell Therapy Ameliorates Pulmonary Fibrosis by Reprogramming Macrophage Activation**

To further assess epithelial stability during co-culture conditions, expression of surfactant-related genes and the ATI marker Aqp-5 was evaluated after 48 h of co-culture with control, BLM-AM or BLM-IM macrophages (Supplementary Fig. S1). Surfactant gene expression showed a general downward trend under co-culture conditions, whereas Aqp-5 expression did not increase above freshly isolated ATII levels, suggesting no evidence of early transition toward an ATI-like phenotype during the experimental period.

***Supplementary Figure S1.*** ***ATII epithelial marker expression during co-culture conditions****. (A) mRNA expression of ATII-associated genes (SP-C, SP-A, SP-B and SP-D) and the ATI-associated marker Aqp-5 in ATII cells after 48 h of co-culture with control-AM or control-IM. (B) mRNA expression of ATII-associated genes (SP-C, SP-A, SP-B and SP-D) and the ATI-associated marker Aqp-5 in ATII cells after 48 h of co-culture with BLM-AM or BLM-IM. Data are expressed as mean ± SD (n = 4). *p < 0.05 vs. freshly isolated ATII cells.*

For fibroblast characterization, α-SMA immunofluorescence staining was performed in explant-derived fibroblast cultures used at passage 3. Nuclei were counterstained with DAPI.

***Supplementary Figure S2. Characterization of explant-derived fibroblast cultures.*** *(A–B) Representative immunofluorescence images of α-SMA (green) and nuclei (blue, DAPI) in fibroblast cultures isolated from control (A) or BLM-treated animals (B) after 48 h of culture. (C) mRNA expression of fibroblast/myofibroblast-associated genes (α-SMA, TGF-β, collagen I, collagen III, FN1 and Ccn2/CTGF) in fibroblast cultures isolated from control or BLM-treated animals after 48 h of culture. Data are expressed as mean ± SD (n = 4). *p < 0.05 vs. control fibroblasts.*

***Supplementary Table S1.*** Primer sequences used for supplementary characterization of ATII cells and fibroblast cultures.

| ***Primers*** | ***Forward*** | ***Reverse*** |
| --- | --- | --- |
| **SP-A** | 5’-CCTGCAGGCTCTGTATGTGG-3’ | 5’-CCCCAGCACCATCTGGTATC-3’ |
| **SP-B** | 5’-TACCCCTGGTGGTGGGTG-3’ | 5’-ACTCAGTGTCTTGCAGTGGC-3’ |
| **SP-C** | 5’-GAAACTCAGAAACGCCTCGC-3’ | 5’-GGCTTATAGGCGGTCAGGAG-3’ |
| **SP-D** | 5’-CTTGGGGCCACCAGGAAG-3’ | 5’-TCTCTGCGAGAGGGTCTTCA-3’ |
| **Aqp5** | 5’-GGGGATCTACTTCACCGGCT-3’ | 5’-CAGCATGGCCCCCACAATA-3’ |
| **TGF-β** | 5’-TGAGTGGCTGTCTTTTGACG-3’ | 5’-TGGGACTGATCCCATTGATT-3’ |
| **α-SMA** | 5’-TGTGCTGGACTCTGGAGATG-3’ | 5’-GAAGGAATAGCCACGCTCAG-3’ |
| **Collagen I** | 5’-AATGGTGCTCCTGGTATTGC-3’ | 5’-GGTTCACCACTGTTGCCTTT-3’ |
| **Collagen III** | 5’-GTCCACGAGGTGACAAAGGT-3’ | 5’-CATCTTTTCCAGGAGGTCCA-3’ |
| **FN1** | 5’-CGAGGTGACAGAGACCACAA-3’ | 5’-CTGGAGTCAAGCCAGACACA-3’ |
| **Ccn2/CTGF** | 5’-ACTGTTGGCGAACAAATGGC-3’ | 5’-CTGCCTCCCAAACCAGTCAT-3’ |
| **GAPDH** | 5’-CTGTGTCTTTCCGCTGTTTTC-3’ | 5’-TGTGCTGTGCTTATGGTCTCA-3’ |
